# Editorial: Sperm epigenetic code: implications in reproductive health and paternal contribution to embryo development

**DOI:** 10.3389/fcell.2025.1666435

**Published:** 2025-09-01

**Authors:** Marta Lombò, Gilda Cobellis, Francesco Manfrevola

**Affiliations:** ^1^ Department of Life and Environmental Sciences, Polytechnic University of Marche, Ancona, Italy; ^2^ Department of Experimental Medicine, University of Campania “Luigi Vanvitelli”, Naples, Italy

**Keywords:** sperm epigenetics, male infertility, fertilization, epigenetic inheritance, offspring health, paternal lifestyle, spermatogenesis

For decades, the study of human reproduction and offspring health has predominantly focused on the maternal contribution to embryo development, often overlooking the molecular complexity of spermatozoa, traditionally considered merely as vectors of paternal genetic information. However, growing evidence reveals that spermatozoa deliver a sophisticated repertoire of epigenetic marks - including DNA methylation patterns, histone post-translational modifications (HPTMs) and RNA cargo - that play crucial roles in defining male reproductive health, embryo development and intergenerational inheritance (Hammoud et al., 2009; Hammoud et al., 2011; Luense et al., 2016; Yamaguchi et al., 2018; Chioccarelli et al., 2020; Martinez et al., 2023). The Research Topic “Sperm Epigenetic Code: Implications in Reproductive Health and Paternal Contribution to Embryo Development” provides multidisciplinary insights into the establishment and maintenance of sperm epigenetic landscape. Since harmful perturbations in the sperm epigenome have been consistently linked to altered sperm quality, male infertility and compromised offspring health, these Research Topic are extensively addressed in this Research Topic.

Dodd and Luense have contributed a review article focused on the role of paternal histones in male reproductive health and early embryogenesis. Despite the genome-wide histone-to-protamines replacement during spermatogenesis, a small subset of histones is retained in mature spermatozoa and marks critical genes potentially involved in early embryo development (Brykczynska et al., 2010; Erkek et al., 2013; Jung et al., 2017; Yoshida et al., 2018). Their review highlights growing evidence that aberrant sperm histone epigenetic marks (particularly histone acetylation and methylation pattern) are associated with multiple male infertility conditions (e.g., asthenoteratozoospermia and asthenozoospermia) as well as impaired embryogenesis, underscoring their importance as key paternal-derived epigenetic regulators. In this context, a crucial determinant for the proper establishment of sperm epigenetic code consists in the chromatin remodeling which arises during spermiogenesis. The hyperacetylation of lysine residues on histone H4 (notably H4K5, H4K8, H4K12, and H4K16) underlie the histone-to-protamine exchange in elongating spermatids. Precise regulation of these histone PTMs is indispensable for the acquisition of a functional paternal epigenome. In this regard, Porreca et al. have provided novel insights into the mechanistic involvement of histone -acetyltransferases (HATs) and -deacetylases (HDACs) in modulating H4 hyperacetylation. By exploiting the cannabinoid receptor 1 (CB1) knockout mice (Cb1^−/−^) as a model of impaired histone displacement, the authors demonstrate how the aberrant interaction between deacetylase SIRT1 and the MOF acetyltransferase disrupts MOF-dependent H4K16 acetylation in elongating spermatids, leading to defective histone displacement and production of spermatozoa with abnormally retained chromatin H3-binding sites.

The sperm epigenetic code is responsive to external environmental and lifestyle stressors such as pollutants, diet, stress and smoking (Chioccarelli et al., 2010; Pastore et al., 2024). In this light, the work of Porreca et al. also clarifies the putative adverse effects on sperm epigenetic landscape deriving from cannabis use. Mounting evidence suggests that alterations in the sperm epigenetic marks, such as DNA methylation, sperm-borne small RNAs, and histone PTMs (particularly acetylation of histones H3 and H4) may arise from metabolic disorders and environmental contaminants and can be transmitted to the following generations (Akhatova et al., 2025; Ma et al., 2020; Lombó et al., 2019; Al Khaled et al., 2018; Hammer et al., 2021; Yuan et al., 2017; Rothstein et al., 2017; Lismer et al., 2020; Terashima et al., 2015; Swanson et al., 2020; Chen et al., 2016). Hence, the study by Pastore et al. sheds light on the intergenerational transmission of dysmetabolic traits via paternal inheritance. Investigating paternal obesity induced by a high-fat diet (HFD), the authors examined how the obesity condition influences sperm quality and offspring health, also assessing the protective potential of antioxidant supplementation with N-acetylcysteine (NAC). While NAC mitigated paternal metabolic dysfunction and improved sperm parameters, it failed to prevent the inheritance of epigenetic alterations lying behind metabolic disorders, as offspring of HFD-fed fathers exhibited persistent metabolic impairments (glucose intolerance and diabetic phenotype) and aberrant DNA methylation at epigenetically controlled loci as *IGFII/H19* and *cyp19a1*. Complementing these findings, Hussain et al. provide a review on the role of redox signaling and metabolism in male reproductive functions, emphasizing the epigenetic responsiveness to toxicant exposure. The documented effects include alterations in sperm DNA methylation and histone H3 methylation patterns, reported to be trans-generationally inherited and capable to impair testicular function and semen quality across multiple generations. The review article by Aljabali et al., unveils how paternal environmental exposures—including stress, high-fat diet, and toxicants—affect sperm-borne epigenetic signals involved in developmental programming of sexual dimorphism. The authors describe a plethora of sperm-derived epigenetic alterations—including disrupted DNA methylation and differential small non-coding RNA (sncRNA) profiles—that contribute to sex-specific developmental anomalies. Notably, these changes arise as early as the preimplantation stage, underscoring the critical role for the paternal epigenome in driving sexually dimorphic developmental trajectories.

As the burden of male infertility continues to rise globally, the urgency to decode the sperm epigenome grows. This emerging Research Topic requires a paradigm shift: moving beyond genetic determinism to embrace a more integrative view of heredity, one that acknowledges the epigenetic memory embedded in the paternal germline. Supporting this notion, Lin et al. provide encouraging evidence that germ cells retain an intrinsic epigenetic memory. The authors demonstrate that although *in vitro* induced pluripotent stem cells (iPSCs) retain residual gene expression and epigenetic features of their source cell type, this memory is largely erased upon their differentiation into primordial germ cell-like cells (PGCLCs), faithfully recapitulating the epigenetic reprogramming triggered *in vivo* during embryonic PGC specification. This study confirms a selective germ cell epigenetic memory and holds great promise for reproductive medicine. In line with the concept of paternal epigenetic memory, Capra et al. performed a comparative study in Montbéliarde and Holstein bull sperm. Despite breed-specific epigenetic variations, the authors reveal the presence of conserved methylated regions across the genetically distinct breeds. Such evolutionary conserved epigenetic signature appears essential for maintaining genomic integrity, silencing transposable elements, and regulating key genes required for spermatogenesis and early embryonic development.

In conclusion, the concept of the sperm epigenetic code radically reshapes our understanding on paternal heredity, extending it beyond DNA sequence to include dynamic, heritable, and environmentally sensitive epigenetic modifications. The vulnerability of sperm epigenome to external stressors, alongside rising global infertility rates, underscores the urgent need to integrate epigenetic knowledge into reproductive medicine and public health strategies. Decoding and ultimately modulating the sperm epigenome offers a promising avenue to improve fertility outcomes and safeguard the health of future generations.
